# Thoracic SMARCA4-deficient undifferentiated tumor

**DOI:** 10.1007/s12672-023-00639-w

**Published:** 2023-04-28

**Authors:** Jiapeng Jiang, Zhixin Chen, Jiali Gong, Na Han, Hongyang Lu

**Affiliations:** 1 The Second Clinical Medical College, Zhejiang Chinese Medical University, Hangzhou, 310053 People’s Republic of China; 2 Zhejiang Key Laboratory of Diagnosis & Treatment Technology on Thoracic Oncology (Lung and Esophagus), Zhejiang Cancer Hospital, Institute of Basic Medicine and Cancer (IBMC), Chinese Academy of Sciences, Hangzhou, 310022 China; 3 Department of Thoracic Medical Oncology, Zhejiang Cancer Hospital, Institute of Basic Medicine and Cancer (IBMC), Chinese Academy of Sciences, Hangzhou, 310022 China

**Keywords:** Prognosis, SMARCA4-UT, Treatment diagnosis

## Abstract

Thoracic SMARCA4-deficient undifferentiated tumor (SMARCA4-UT) is a recently described smoking-related malignancy. The pathogenesis of SMARCA4-UT is the mutational inactivation and loss of expression of a subunit encoding the mammalian switch/sucrose nonfermenting ATPase-dependent chromatin remodeling complex (which can be mobilized using adenosine triphosphate hydrolysis nucleosomes and regulate other cellular processes including development, differentiation, proliferation, and apoptosis), in particular SMARCA4 and SMARCA2. The dynamic activity of this complex plays an important role in regulating the activation and repression of gene expression programs. SMARCA4-UT exhibits morphological features similar to the malignant rhabdoid tumor (MRT), small cell carcinoma of the ovary of the hypercalcemic type (SCCOHT), and INI1-deficient tumor, but SMARCA4-UT differs from SCCOHT and MRT from a genomic perspective. SMARCA4-UT mainly involves the mediastinum and lung parenchyma, and appears as a large infiltrative mass that easily compresses surrounding tissues. At present, chemotherapy is a common treatment, but its efficacy is not clear. Moreover, the inhibitor of the enhancer of zeste homolog 2 showed promising efficacy in some patients with SMARCA4-UT. This study aimed to review the clinical characteristics, diagnosis, treatment, and prognosis of SMARCA4-UT.

## Introduction

In 2015, Le Loarer et al. [1] identified a group of undifferentiated rare thoracic malignancies and named them SMARCA4-DTS. SMARCA4-DTS was listed as a newly described solid tumor in the fifth edition of the *WHO Classification of Thoracic Neoplasms* [2]. The name was changed to SMARCA4-UT in the 2021 *WHO Classification of Thoracic Tumors*. SMARCA4-UT is primarily characterized by the loss of the SMARCA4 gene, which is located on chromosome 19p and encodes the Brahma-related gene 1 protein, one of the two mutually exclusive catalytic subunits of the switch (SWI)/sucrose nonfermenting (SNF) chromatin remodeling complex [3–5]. The frequency of mutated SWI/SNF subunits is high in a variety of human tumors and developmental diseases, with mutations in SWI/SNF subunits or associated proteins present in approximately 20% of human cancers [6–8]. The SWI–SNF complex plays an important role in transcription, replication, DNA repair and recombination, regulation of gene expression, and cell cycle regulation [9–11]. Moreover, Le Loarer et al. [1] found that SMARCA4-UT was genetically different from SMARCA4-deficient non-small-cell lung cancer (NSCLC) and exhibited morphological features more similar to high-boundary small-cell carcinoma of the ovary of the hypercalcemic type (SCCOHT) and malignant rhabdoid tumor (MRT). The clinical manifestations of SMARCA4-UT are not unique and difficult to distinguish from other tumors. Therefore, the correct diagnosis of SMARCA4-UT requires a combination of clinical manifestations, laboratory tests, histopathology, immunohistochemistry, and mutational analysis. Currently, comprehensive treatment such as chemotherapy, radiotherapy, and targeted therapy is mainly used. In recent years, studies have found that epigenetic changes, such as DNA methylation, histone modification, and noncoding RNA expression, play an essential role in cancer development. The SWI–SNF complex has become a new class of tumor suppressor genes; therefore, epigenetic therapy may also become a new treatment modus operandi for SMARCA4-UT [12].

### Clinical characteristics

SMARCA4-UT is a recently reported very rare type of thoracic tumor. Epidemiologically, SMARCA4-UT occurs mostly in the middle ages (median age at presentation 58 years, age range 27–90 years). Research showed that most patients were men and had a smoking history (more than 90%). Also, no studies showed how SMARCA4-UT was related to race or geography [2]. SMARCA4-UT exhibits no unique clinical manifestations, and visible clinical symptoms include dyspnea, chest pain, regurgitation, fatigue, and weight loss; when the pleura invades, it can manifest as recurrent pleural effusion or pleural empyema [2, 3]. SMARCA4-UT presents as an invasive mass, often invading adjacent organs. At diagnosis, SMARCA4-UT is predominantly located in the mediastinum, followed by the pleura and lung, and can compress and penetrate adjacent organs, resulting in superior vein syndrome, atelectasis, spinal cord compression, and esophageal invasion. Studies showed that many patients died of local complications [3, 13]. Perret et al. [14] found that metastatic diseases were prevalent. Usually, they occurred in the affected sites in the following order: lymph nodes, bones, adrenal glands, liver, gastrointestinal tract, central nervous system, and kidney [14]. Moreover, Nambirajan et al. [15] found SMARCA4-UT incidentally in the soft tissue of the chest wall around the site of implantable cardioverter defibrillator insertion in a patient with chronic empyema of indeterminate etiology for more than 10 months. The findings of this case suggested a possible link between chronic infection/inflammation and SMARCA4-UT. To date, any link between chronic infection/inflammation and SMARCA4-UT could not be established [15]. Kunimasa et al. [16] reported two patients with SMARCA4-UT with severe bone metastases, which led them to hypothesize that severe skeletal-related event (SRE) might be a new important clinical feature of SMARCA4-UT. However, this postulation needs further verification.

### Imaging examination

Computed tomography (CT) and positron emission tomography CT (PET-CT) are important and basic methods for detecting SMARCA4-UT. Nambirajan et al. [2] systematically reviewed the CT results of 21 patients with SMARCA4-UT and found that most of them presented as mediastinal mass involving the upper and middle mediastinum, and more than two thirds of the cases exhibited continuous lung parenchyma involvement. A small proportion of patients with SMARCA4-UT developed unilateral, unilateral, or multifocal masses involving visceral and parietal pleura. Moreover, no calcification or cystic changes were found [2]. SMARCA4-UT is heterogeneous in CT. SMARCA4-UT is more likely to cause vascular coating than radiosensitive neoplastic lymphomas [17]. Almost all SMARCA4-UT showed aggressive and dilated growth, mainly in the mediastinum and unilateral pleura (although mediastinal structures were extensively involved, most SMARCA4-UT came from the lung), with the involvement of thoracic and cervical lymph nodes. Furthermore, the tumor often showed marked necrosis. Briefly, SMARCA4-UT should be suspected if the chest mass is large, heterogeneous, and exhibits infiltration and compression of surrounding tissues, accompanied by necrotizing lymphadenopathy [2, 13, 18]. SMARCA4-UT and its metastases always showed strong 18F-fluorodeoxyglucose affinity [13]. However, different thresholds for positivity in PET scans may alter diagnostic performance. A strict positive threshold reduces sensitivity and increases specificity [19]. Considering its metastatic tendency and metabolic activity, the clinical staging classification of SMARCA4-UT should include PET-CT and CT [13].

### Pathology and molecular characteristics

SMARCA4-UT histologically resembles SMARCB1 (INI1)-deficient tumors, showing a highly rhabdoid appearance and frequent cytokeratin expression in tumor cells [3]. Cytological smears showed many atypical round or polygonal cells (epithelioid cells) in a single or loose distribution. SMARCA4-UT cells exhibited well-defined boundaries, sparse to medium cytoplasm, enlarged vesicular nuclei, and prominent nucleoli. Moreover, some SMARCA4-UT cells were observed to exhibit nuclear eccentricity and eosinophilic cytoplasm [7, 20–23]. SMARCA4-UT is highly malignant and exhibits some necrosis areas [24].

In terms of immunohistochemistry, Crombé et al. [13] showed that most of the tumors in the study cases lost SMARCA4 gene expression. Among 19 patients, 17 (89.5%) were positive for sex-determining region Y–box 2 (SOX2) and 16 (76.2%) were positive for CD34 (a transmembrane phosphoglycoprotein encoded by the CD34 gene). Moreover, markers for breast adenocarcinoma and lung adenocarcinoma were negative [13]. In another study, Anžičn et al. [9] also found that tumor cells showed loss of SMARCA4 expression, diffuse expression of CD34, and weak focal expression of epithelial membrane antigen (EMA) and CD99. Stewart et al. [24] reported a complete loss of SMARCA4 and SMARCA2 reactivity in tumors. INI1 was completely preserved, and ROS1 IHC results were negative. At the same time, CK7, AE1/3, Cam5.2, and SALL4 showed focal responses, while CD34 and SOX2 showed sporadic responses. Hence, immunohistochemical screening using multiple antibodies including SMARCA4 and SOX2 is necessary to diagnose SMARCA4-UT [16, 25]. Useful markers for diagnosis include immunopositivity for EMA, CD34, SALL4, and SOX2, and loss of expression of SMARCA2 [26, 27]. In addition, Leckey et al.[26] found that p40 and p63 were rarely expressed in SMARCA4-UT. The immunohistochemical results of SMARCA4-UT are shown in Table 1 [5, 9, 14, 15, 17, 23–26, 28–30].

**Table 1:** Immunohistochemical results of SMARCA4-UT examination

Refs	No. of patients	IHC results										
		P40	Claudin4	CD99	SALL4	AE1/3	CD34	EMA	SMARCA2 deficiency	SOX2	INI-1	SMARCA4 deficiency
[5]	2	NM	–	+	+	+	+	NM	+	+	NM	+
[9]	1	NM	NM	+	NM	NM	NM	+	NM	NM	+	+
[14]	30	0/30 +	2/19 +	4/15 +	7/21 +	NM	17/27 +	NM	30/30 +	26/27 +	23/23 +	30/30 +
[15]	1	–	NM	NM	+	+	+	–	NM	NM	+	+
[17]	1	NM	NM	NM	NM	NM	NM	NM	–	NM	NM	+
[23]	1	–	–	NM	NM	–	+	+	NM	NM	NM	+
[24]	1	NM	NM	NM	+	+	+	NM	+	+	+	+
[25]	1	NM	–	NM	NM	+	+	+	+	+	NM	+
[26]	1	NM	–	+	NM	–	+	+	NM	NM	+	+
[28]	1	NM	NM	NM	+	–	+	–	NM	+	NM	+
[29]	1	–	–	NM	+	+	–	NM	+	+	+	+
[30]	1	NM	NM	NM	NM	NM	NM	NM	–	NM	NM	+
Total( +)	42	0/33	(2/24)	(6/18)	(12/26)	(5/8)	(23/35)	(4/6)	(34/36)	(32/33)	(28/28)	(42/42)
Rate	/	0% +	8% +	33% +	37% +	62% +	65% +	67% +	94% +	97% +	100% +	100% +

+  positive, – negative, *NM* not mentioned

In recent years, studies have found that epigenetic changes play an important role in cancer development. Almost all cancers exhibit epigenetic changes, such as hypomethylation of oncogenes, hypermethylation of tumor suppressor genes, depletion of hydroxymethylation, changes in histone acetylation and methylation patterns, and changes in miRNA expression levels [31]. The SMARCA4 gene in the SWI/SNF complex encodes the transcription-activating protein BRG1, which recognizes acetylated lysine residues, such as those in the tail of the n-terminal histone; These domains play a key role in regulating gene transcription [32]. In addition, SMARCA4 deletions affect the level of H3K27 acetylation and alter nucleosome localization, thus regulating transcription [33, 34]. In terms of PTMs, PTMs can recruit reading proteins that specifically bind to different PTMs or their combinations and regulate downstream events, or regulate histone binding to DNA and interactions between adjacent nucleosomes, thus leading to genomic instability by altering chromatin structure. For example, SWI/SNF complexes can regulate the degree of DNA encapsulation by mobilizing and reshaping nucleosome structure by sliding and catalyzing histone octamer. When DNA is tightly packed, gene expression is inhibited. Moreover, SWI/SNF complexes recruit histone deacetylases (HDACs) to remove activated acetyl markers from histone tails, thereby inhibiting tumor cell cycle [28, 32, 35]. Not only that, part of the protein encoded by the mutated gene may be the regulatory factor of histone PTMs, tumor suppressor genes may be silenced by changes in PTMs level, and oncogenes may be activated by changes in PTMs level [35]. In addition, some common epigenetic changes in cancer play a role in the development and development of lung cancer. For example, methylation can silence gene expression by interfering with transcriptional mechanisms, Recombinant DNA Methyltransferase 1 (DNMT1) is the main enzyme that maintains methylation patterns after DNA replication. DNMT1 is highly expressed in lung cancer cells, and methylates and silenced cell cycle regulatory factors, such as p16 and p53. But whether these epigenetic changes occur in SMARCA4-UT is not clear [31]. In conclusion, disruption of SWI/SNF function and deletion of SMARCA4 genes can lead to changes in chromatin structure that affect gene expression and disrupt differentiation procedures, exhibiting "epigenetic instability" that ultimately leads to cancer formation [28]. Moreover, in cancer, genetic and epigenetic processes interact, such as gene mutations resulting from changes in histone modification sites and increased mutation rates when chromatin structure is altered [35].

**Fig. 1:**
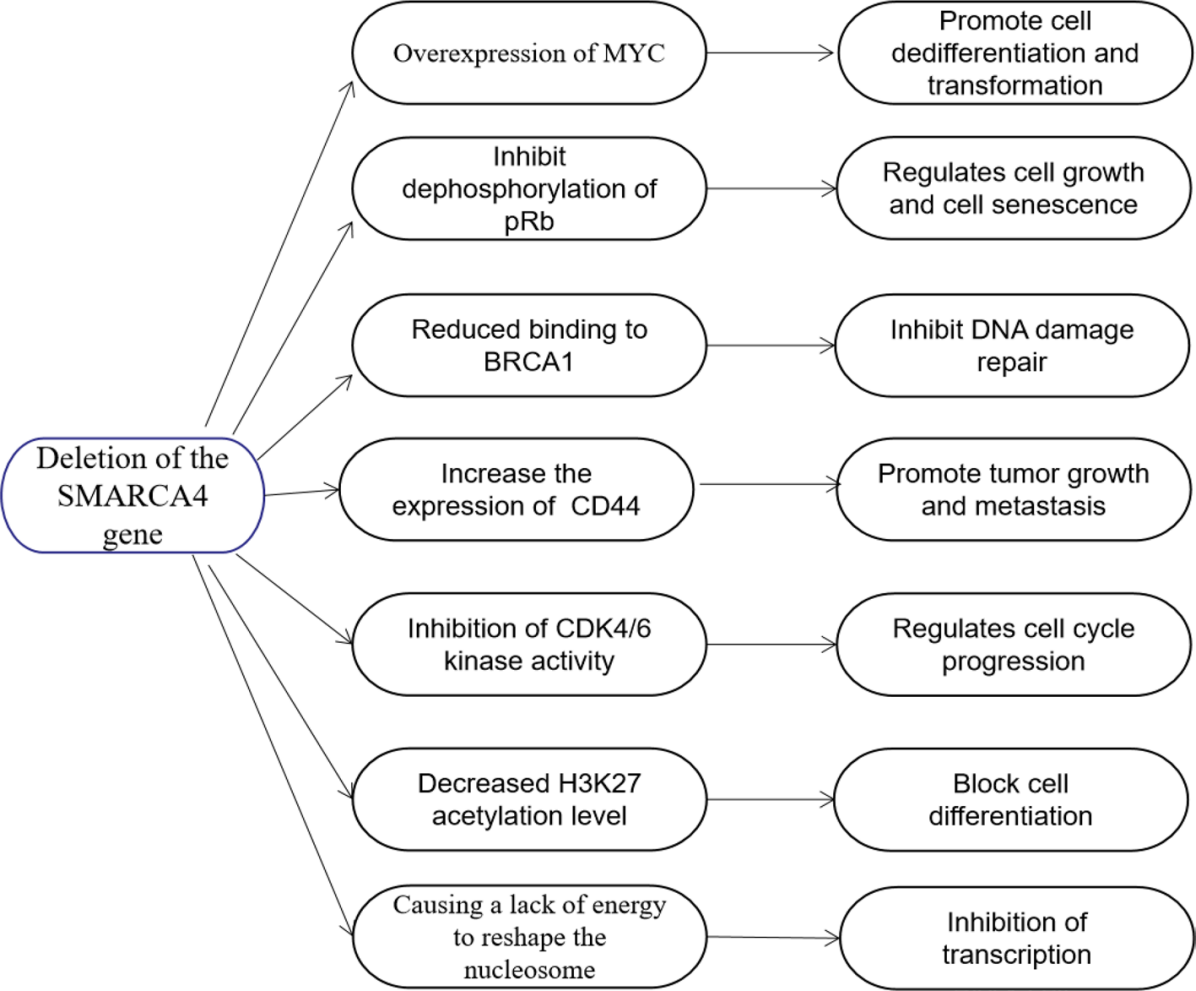
Molecular pathways that may be affected by SMARCA4 loss

The SWI/SNF chromatin remodeling complex uses the energy of ATP hydrolysis to reshape nucleosomes and regulate transcription. There is growing evidence that SMARCA4 genes and SWI/SNF complexes play a broad role in tumor inhibition, and SMARCA4-UT may cause many molecular pathways to be affected. The SWI/SNF complex can interact with MYC, and SMARCA4 can fight MYC by binding to MYC itself or any promoters of MYC. Loss of SMARCA4 can lead to overexpression of MYC and expression of undifferentiated genes, which can affect cell cycle progression [36]. In addition, SMARCA4 is also involved in dephosphorylation of pRb and up-regulation of the CDK inhibitor p21, leading to downregulation of various components of the pRb cascade, thereby regulating cell growth and cell aging [37]. SMARCA4 encodes the expression of BRG1 protein, which activates or inhibits transcription by regulating the ATPase of the chromatin remodeling complex. The protein also binds to BRCA1 (a gene product critical for DNA damage repair) and regulates the expression of the tumorigenic protein CD44, which is involved in the growth and metastasis of a variety of tumors [38, 39]. Loss of SMARCA4 also leads to downregulation of CD1, limits CDK4/6 activity in tumor cells, and affects sensitivity to CDK4/6 inhibitors [40]. The possible molecular pathways affected by SMARCA4 loss are shown in Fig. 1.

Recent studies have found surprisingly high frequencies of mutated SWI/SNF subunits in various human tumors and developmental diseases [6, 12]. Further, Nambirajan et al. [2] found that SMARCA4-UT exhibited significant genomic overlap with SMARCA4-deficient NSCLC, although its gene expression profile was different from that of SMARCA4-deficient NSCLC. They had frequent mutations in TP53, STK11, KEAP1, and KRAS [2]. Rekhtman et al. [18] sorted out 18 patients with SMARCA4-UT and found that 50% patients (9/18) had typical NSCLC gene mutations, including STK11 (*n* = 6), KEAP1 (*n* = 4), and KRAS (*n* = 5) mutations. In one of the patients, KRAS, STK11, and KEAP1 were found mutated [18]. Liu et al. [41] found that conventional drug therapy did not respond well in patients with both KRAS and SMARCA4 mutations. The frequency of mutations in SMARCA4-UT is shown in Table 2 [1, 14, 16, 18, 24, 25, 29, 42, 43].

**Table 2:** The frequency of gene mutations in SMARCA4-UT

Refs.	No. of patients	Gene mutation									
		SMARCA4	TP53	CDKN2A	STK11	KRAS	KEAP1	ARID1A	ALK	EGFR	ROS1
[1]	13	13	9	NM	NM	NM	NM	NM	NM	NM	NM
[14]	2	2	2	1	0	1	1	1	0	0	0
[16]	2	2	2	0	1	0	0	0	1	0	0
[18]	18	18	16	NM	6	5	4	NM	NM	NM	NM
[24]	1	1	1	0	NM	0	0	0	NM	NM	NM
[25]	1	1	1	NM	NM	NM	NM	NM	NM	NM	NM
[29]	1	1	1	1	NM	NM	NM	NM	0	0	0
[42]	5	5	5	2	NM	1	1	NM	NM	NM	NM
[43]	1	1	1	NM	NM	NM	NM	NM	NM	NM	NM
Total	44	44	38/44	4/11	7/22	7/28	6/28	1/5	1/15	0/5	0/5
Rate	/	100%	86.4%	36.4%	31.8%	25%	21.4%	20%	6.7%	0%	0%

*NM* not mentioned

### Diagnosis and differential diagnosis

Since the clinical presentation of SMARCA4-UT is not unique, a combination of laboratory tests, histopathology, and molecular features is required to diagnose SMARCA4-UT. Pathology is the gold standard for SMARCA4-UT diagnosis, and the diagnosis of SMARCA4-UT usually requires the following three conditions: (1) rhabdoid or poorly differentiated phenotype; (2) complete or major loss of SMARCA4 and SMARCA2 expression; (3) focal or diffuse expression of at least two species of the following markers: SOX2, CD34, and SALL4 [24]. The tumor mutation burden of SMARCA4-UT was comparable to that of SMARCA4-deficient NSCLC (14.2 and 15.8 mutations per megabase, respectively). Furthermore, SMARCA4-UT and SMARCA4-deficient NSCLC exhibited the same overt smoking characteristics and smoking-related NSCLC type mutations (KRAS, STK11, and KEAP1) [18, 44, 45]. Therefore, distinguishing SMARCA4-deficient NSCLC from SMARCA4-UT is important for the treatment and prognosis of SMARCA4-UT. SMARCA4-deficient NSCLC is usually excluded on the basis of morphology because it exhibits better histological differentiation as clear adenocarcinoma or very rare squamous cell carcinoma morphology [2, 46]. However, differentiation between SMARCA4-deficient NSCLC and SMARCA4-UT can be difficult or sometimes impossible in microscopic biopsies because of poor morphology, immunosignature abnormalities, or sarcomatoid carcinoma in the biopsied region [2]. Le Loarer et al. [1] found that SMARCA4-UT was often accompanied by the loss of SMARCA2, whereas SMARCA4-deficient NSCLC was usually not accompanied by the loss of SMARCA2, and CD34 was less expressed in SMARCA4-deficient NSCLC [47]. One of the most striking differences is that the tumor size of SMARCA4-UT is significantly larger than that of SMARCA4-deficient NSCLC [18].

Recent studies have reported that SMARCA4-UT cannot be diagnosed morphologically because it exhibits morphological features similar to MRT, SCCOHT, and INI1-deficient tumors. Furthermore, both SMARCA4-UT and SCCOHT exhibit an aggressive clinical course and have similar SMARCA4 mutational patterns. However, from a genomic perspective, SMARCA4-UT differs from SCCOHT and MRT [22]. SMARCA4-UT differs from MRT, and MRT tends to be present in young children [42, 43]. SCCOHT displays unique immune features, including the expression of Wilms’ tumor suppressor gene 1 (WT1), EMA, vimentin, and cytokeratin [48].

Kunimasa et al. [16] analyzed and revealed some histopathological features associated with sarcomas in two patients. Both patients showed loss of claudin-4 expression, which distinguished SMARCA4-UT from small round cell tumors and rhabdomyoid cell tumors [16]. Compared with other tumors, large cell lymphoma can be ruled out by negative expression of lymphoid markers. Negative S-100 immunohistochemistry can also rule out malignant peripheral nerve sheath tumors and melanoma [28]. The negative expression of the epithelial markers CK-AE1/AE3, pan-CK, and EMA can exclude anaplastic carcinoma [28]. HepPar-1 expression in lung adenocarcinomas is generally diffuse and intense, with distinctive granules (mitochondria) [49]. EMA, vimentin diffuse strong positive epithelioid sarcoma and SMARCA4 defective soft tissue tumors were also excluded. Moreover, epithelioid sarcomas consist of epithelioid and spindle tumor cells arranged in cell nodules, with occasional central necrosis and pseudogranulomatous appearance [26, 28, 50]. Differentiation can also occur from gastrointestinal stromal tumors via the detection of CD117 and DOG-1. Negative CDK4 and MDM2 can also exclude the diagnosis of liposarcoma [28]. Furthermore, unlike SMARCA4-UT, which is often associated with heavy smoking, SMARCA4-deficient uterine sarcoma is not associated with smoking [51]. Finally, the distinction between SMARCA4-UT and epithelioid angiosarcoma is also challenging, since the latter is mostly CD34 positive. Because epithelioid angiosarcoma exhibits red blood cells within the cells, intracytoplasmic vacuoles within the cells, and is diffusely positive for CD31, this may help diagnose angiosarcoma [43].

In conclusion, when both SMARCA4 and SMARCA2 are absent, SMARCA4-UT is highly specific for all other tumors that may undergo differential diagnosis [21]. When combined with the frequent co-occurring positivity of CD34, SALL4, and SOX2 and the lack of diffuse expression of epithelial markers, SMARCA4-UT needs to be strongly suspected [15]. The study by Crombé et al. also highlighted a propensity for lymphatic spread, a rare feature of sarcoma that may help establish diagnostic hypotheses [13]. Further, Kuwamoto et al. [43] observed that the interpretation of SMARCA4 immunostaining is not always straightforward. If other clinicopathological findings strongly suggest the diagnosis of SMARCA4-UT, genetic analysis may be considered for further diagnosis [43].

### Treatment

At present, no uniform standard exists for the treatment of SMARCA4-UT. Common treatment scheme includes surgery, chemotherapy, radiation therapy, targeted therapy, immunotherapy and epigenetic therapy. Surgical intervention or combined chemotherapy can be used [14]. Le Loarer et al. [1] documented two patients who underwent surgery; the margin of tumor tissue was negative after surgery, but the tumor still regenerated within 1 month. Decroix et al. [52] found that part of the tumor could be removed surgically; however, the tumor recurred in less than a month. Hence, SMARCA4-UT has a high degree of malignancy and is prone to relapse. Patients are often in the late stage when they are diagnosed, indicating difficulty in achieving the desired effects of surgery. At present, chemotherapy is the most commonly used treatment. The common chemotherapy regimen is paclitaxel + carboplatin; other commonly used drugs include gemcitabine, cisplatin, and doxorubicin. However, the effect of chemotherapy is unclear due to the lower prevalence rate of SMARCA4-UT, and the survival time of patients after chemotherapy is often less than 1 year.

In terms of targeted therapy, the inhibitor of the enhancer of zeste homolog 2 (EZH2) is currently promising for treating SMARCA4-UT [28]. EZH2 is a histone methyltransferase that serves as the catalytic subunit of the polycomb repressor complex (PRC), a transcriptional regulator involved in cancer metastasis and cell proliferation. The SWI–SNF complex regulates PRC, and if this regulation is perturbed by mutations in the SWI–SNF complex protein, uninhibited PRC activity can lead to tumor progression and metastasis [3, 53, 54]. Inhibiting its methyltransferase activity using an EZH2 inhibitor reduces H3K27me3 levels, thereby rebalancing transcriptional activity. Tazemistat, an inhibitor of EZH2 activity, is currently being evaluated in a phase II study [52]. Data from a phase I clinical trial of EZH2 inhibitors in INI1-negative or SMARCA4-negative solid tumors have shown encouraging results, with some patients achieving complete or partial remission, and some patients remaining in stable disease conditions for more than 2 years [26]. Kim et al. [55] found that SWI/SNF mutant tumors were only partially dependent on EZH2 histone methyltransferase activity, suggesting that currently clinically developed EZH2 inhibitors might not fully inhibit the oncogenic activity of EZH2. Notably, while patients may not respond to EZH2 inhibitors as a single agent, they may respond to a combination of EZH2 and BCL2 inhibitors [56]. Moreover, in the study of CDK4/6 in glioblastoma multiforme, Wiedemeyer et al. [57] found that the functional status of cancer-related pathways and specific changes in the molecular network of pathway members were key modifiers of the response to targeted therapy.

In terms of immunotherapy, Anžič et al. [9] administered patients with SMARCA4-UT eight cycles of pembrolizumab, which resulted in the progression of cervical lymph node disease and mediastinal lymph node regression. Subsequently, the patient received four cycles of pembrolizumab and ipilimumab, and showed mixed responses to the drugs [9]. Coincidentally, Nambirajan et al. [21] described a patient with SMARCA4-UT who partially responded to immunotherapy with pembullimab and ipilimumab, and who was still alive 22 months after diagnosis. Iijima and Takada et al. [29, 30] found that cytotoxic chemotherapy as the first- and second-line treatment did not show sufficient therapeutic effect, but the third-line treatment using nivolumab showed significant tumor regression. Lijima et al. described a patient who was treated with nivolumab as the third-line therapy, resulting in a dramatic and almost undetectable reduction in tumor size [29]. Jelinic et al. [58] sorted out the data and found that SMARCA4-regulated transcriptional programs might affect tumor immunogenicity, leading to tumor-infiltrating lymphocyte infiltration and upregulation of programmed cell death ligand 1 (PD-L1), indicating the significant response of SMARCA4-UT to anti-PD-1 immunotherapy.

In terms of epigenetic therapy, SMARCA4-UT is closely related to histone epigenetic modification, and drugs such as histone deacetylase and histone methyltransferase inhibitor may play an important role in SMARCA4-UT therapy through epigenetic regulation [31]. In addition, enzymes that modify chromatin structure may also be involved in the occurrence and development of tumors, and the epigenetic changes may be reversible, and inhibition of chromatin modification enzymes may have important therapeutic significance for cancer [28].

The treatment regimen for SCHOOT is also expected to improve the prognosis of SMARCA4-UT due to the similarity between SMARCA4-UT and rhabdomidal tumors [59]. Lissanu Deribe et al. [60] found that gene expression analysis revealed enhanced oxidative phosphorylation (OXPHOS) features in SMARCA4 mutant tumors. These findings provided a mechanistic basis for further development of OXPHOS inhibitors as therapeutic agents for SWI/SNF mutant tumors [60]. Hence, the current first- and second-line treatments still involve mainly chemotherapy, supplemented by radiotherapy, but the prognosis is not significantly improved. Recently, immunotherapy (pembrolizumab, ipilimumab, and nivolumab) and targeted therapy (EZH2 inhibitors) have shown promising results. The treatment and prognosis of SMARCA4-UT patients are shown in Table 3 [5, 9, 16, 17, 22, 26, 28–30].

**Table 3:** Clinical characteristics, treatment, and outcomes of patients with SMARCA4-UT

Refs	No. of patients	Age	Sex	Treatment	Metastasis	Tumor location	OS (month)	Result
[5]	1	70	Male	CT	NM	Pleura	11	Death
[5]	1	50	Male	CT + RT	Cervical lymph node	Mediastinum Pleura	1	Death
[9]	1	41	Male	T&C + ICIs (pembrolizumab) + H	Neck	Mediastinum	24	Death
[16]	1	45	Male	T&C + ICIs	Bone	NM	11	Death
[16]	1	45	Male	T&C + ICIs	Bone	NM	11	Death
[17]	1	39	Male	RT (40 Gy/20 Fr)	Pulmonary artery	Mediastinum	2	Death
[22]	1	30	Female	H	Axillary lymph node	Pleura	NM	Survival
[26]	1	63	Male	H&G/D	Skin, abdomen	NM	5	Death
[28]	1	44	Male	G&P	Abdomen, bilateral	Mediastinum	6	Death
[29]	1	76	Male	T&C + ICIs, C + VP-16	neck, mediastinal Lymph nodes	Mediastinum	> 22	Survival
[30]	1	69	Female	ICIs (pembrolizumab)	Skin,breast peritoneum	Mediastinum	NM	Survival

*C* Carboplatin, *CT* chemotherapy, *D* docetaxel, *G* gemcitabine, *H* doxorubicin, *NM* not mentioned, *P* cisplatin, *RT* radiation therapy, *T* paclitaxel, *VP-16* etoposide

### Prognosis

SMARCA4-UT is an aggressive tumor that metastasizes rapidly; therefore, early diagnosis is critical for prognosis. Sauter et al. [3] evaluated the prognosis of 37 patients with thoracic tumors, including 8 patients with SMARCA4-UT. The median survival time and 2-year survival rate were significantly lower in patients with SMARCA4-UT than in patients with undifferentiated thoracic tumors that retained SMARCA4 (mOS: 4 vs 39.9 months; 2-year survival rate: 12.5% vs 64.4%). They concluded that SMARCA4-UT exhibited a worse prognosis than undifferentiated thoracic tumors that retained SMARCA4 [3]. Hence, SMARCA4-UT has a very poor prognosis, with a current average survival time of only 6–7 months.

## Conclusions

SMARCA4-UT is a recently identified rare malignancy. Most patients exhibited a history of heavy smoking and lung diseases, such as pulmonary bullae or emphysema. SMARCA4-UT is more likely to occur in the mediastinum and less likely to occur in the pleura. SMARCA4-UT often presents with loss of SMARCA4 and SMARCA2 (which can be distinguished from SMARCA4-deficient NSCLC), but a small number of SMARCA4-UT retain SMARCA2. The IHC data for some patients with SMARCA4-UT are presented in Table 1. Not all SMARCA4-UT exhibited the loss of SMARCA2, and SMARCA4-UT often expressed SOX2. The morphology of SMARCA4-UT was found similar to that of MRT, SCCOHT, and INI1-deficient tumors. However, from a genomic perspective, SMARCA4-UT was distinct from SCCOHT and MRT. MRT is largely restricted to infants and young children and is often associated with germline mutations in related genes that are not characteristic of SMARCA4-UT. SCCOHT exhibits unique immune features, including expression of WT1, EMA, vimentin, and cytokeratin. As shown in the Table3, commonly used chemotherapy agents include paclitaxel, carboplatin, and gemcitabine, and the survival time after chemotherapy is usually less than 1 year. Some patients with SMARCA4-UT responded to ipilimumab and pembrolizumab during immunotherapy, and nivolumab also showed tumor regression in some patients with SMARCA4-UT. Moreover, data from phase I clinical trials of EZH2 inhibitors in INI1-negative or SMARCA4-negative solid tumors showed encouraging results, with some patients achieving complete or partial responses and some remaining stable for more than 2 years. However, most patients had a poor prognosis because of the rapid deterioration and progression of the disease, with a median survival time of only 6–7 months. Understanding the affected molecular pathways in SMARCA4-UT may be of great help in improving the prognosis of SMARCA4-UT.

## Data Availability

Not applicable.
